# Editorial: Metabolite and Nutrient Transporters in Cancer-Cell Metabolism: Role in Cancer Progression and Metastasis

**DOI:** 10.3389/fcell.2022.885717

**Published:** 2022-04-25

**Authors:** Vadivel Ganapathy, Sebastian Haferkamp, Eric K. Parkinson, Maria E. Mycielska

**Affiliations:** ^1^ Department of Cell Biology and Biochemistry, Texas Tech University Health Sciences Center, Lubbock, TX, United States; ^2^ Department of Dermatology, University Hospital Regensburg, Regensburg, Germany; ^3^ Centre for Immunobiology and Regenerative Medicine, Queen Mary University of London, London, United Kingdom; ^4^ Department of Structural Biology, Institute of Biophysics and Physical Biochemistry, University of Regensburg, Regensburg, Germany

**Keywords:** cancer-cell metabolism, nutrient/acid/base transporters, Warburg effect, metabolomics, citrate, carnitine, amino acids, glucose

## Introduction

There has been a noticeable surge of interest in cancer-cell metabolism. In order to support their specific metabolic needs and rapid proliferation, cancer cells not only enhance the rates of normal metabolic pathways but also short-circuit and/or substantially modify other metabolic pathways (Ganapathy-Kanniappan, 2018; Kubicka et al., 2021; Vaupel and Multhoff, 2021; Pavlova et al., 2022). These changes are carried out through induction or suppression of specific enzymes and also through mutations in particular enzymes. As a result, terms such as Warburg effect, aerobic glycolysis, glutamine addiction, glutaminolysis, reductive carboxylation, oncometabolites, methionine-serine-one-carbon pathway, and ferroptosis have become routine in the vocabulary related to cancer biology. The relatively recent identification of cell-surface G-protein-coupled receptors for metabolites such as lactate and the ketone body β-hydroxybutyrate also tie into cancer-cell metabolism (Ristic et al., 2017; Brown and Ganapathy, 2020). These new developments in the cancer field are likely to result in identification of novel, hitherto unrecognized, drug targets for cancer treatment. Surprisingly however, as exciting as these new discoveries are in cancer-cell metabolism, the fact that enhanced entry of selective nutrients and metabolites into cancer cells is the first upstream event in driving these metabolic pathways has not received its due recognition and attention. This was the impetus for the Special Issue in Frontiers in Cell and Developmental Biology to focus on metabolite and nutrient transporters in cancer-cell metabolism.

### Contributions to the Research Topic

The key metabolites/nutrients that are central to the above-mentioned cancer-cell-specific metabolic pathways are glucose, amino acids, fatty acids, lactate, citrate, cholesterol, carnitine, and iron. Metabolism of glucose in cancer cells is related to Warburg effect, aerobic glycolysis, endogenous synthesis of serine for the one-carbon pathway, and provision of the starting substrate (glucose-6-phosphate) for the pentose-phosphate pathway. The article by Shin and Koo focuses on the role of the facilitative glucose transporter GLUT1 (SLC2A1) in breast cancer how this transporter and glycolysis-associated enzymes are upregulated in cancer cells, and how the resultant enhanced glucose delivery into cells feeds into specific pathways to generate lactate, serine, and NADPH. When the rate of glycolysis is enhanced in cancer cells, the intermediates in the pathway are present at higher than normal levels. The first intermediate is glucose-6-phosphate that can be siphoned off into the pentose-phosphate pathway to generate NADPH, a critical component of the anti-oxidant machinery essential for cancer-cell survival. 3-Phosphoglycerate, another intermediate, is used to synthesize serine, the major carbon source for the one-carbon metabolism that is essential for purine/pyrimidine synthesis, methionine-homocysteine cycle, and maintenance of epigenetic landscape. Recent studies have however shown that the increased glucose entry into cancer cells might not be solely due to the upregulation of GLUTs; Na^+^-coupled glucose transporters SGLT1 and SGLT2 are also expressed at increased levels in certain cancers and aid in the successful use of PET scan-dependent diagnosis of cancers (Sala-Rabanal et al., 2016). The review by Nałęcz is related to the amino acid transporter SLC6A14, which is upregulated in several cancers such as pancreatic cancer, breast cancer, colon cancer, and ER-positive breast cancer (Sniegowski et al., 2021). This transporter is broad-specific with ability to transport 18 of the 20 proteinogenic amino acids coupled to multiple driving forces (Sikder et al., 2017). This article details the trafficking pathway for the newly synthesized SLC6A14 protein across the Golgi and the relevance of cancer-related signaling pathways to the increased cell-surface expression of the transporter in cancer cells. The article by Nunes et al. is on cysteine. Generally, this amino acid is discussed in the cancer field primarily in relation to its role in glutathione synthesis and the resultant protection against oxidative stress and ferroptosis (Koppula et al., 2021). But, Nunes et al. focus on the role of cysteine as an energy substrate for cancer cells. In this article, they outline not only the transport pathway for acquisition of extracellular cystine via the transporter xCT (SLC7A11) as a source of cellular cysteine but also the endogenous synthetic pathway via cystathionine.

Fatty acids are energy-rich nutrients with the highest caloric value among the major nutrients. Normoxic cancer cells with capacity for oxidative metabolism use fatty acids via mitochondrial fatty acid oxidation to generate energy. This is the topic for the article by Console et al. The delivery of fatty acids from the cytoplasm into mitochondria across the inner mitochondrial membrane is obligatorily dependent on the metabolite/nutrient carnitine. The review by Console *et al* details the various transporters for carnitine in terms of their expression and function in cancer cells.

Most cancer cells function as if they are under hypoxic conditions even when they are not. Hypoxic environment is common for cancer cells that grow far away from blood vessels. But, even in those cells that are present close to blood vessels, metabolites such as lactate, succinate, and fumarate stabilize the hypoxia-inducible factor-1α by inhibiting prolyl hydroxylases to facilitate hypoxic metabolism. The result is the generation of lactate as the end product of glycolysis and the process is called aerobic glycolysis. The article by Sun et al. describes the monocarboxylate transporters MCT1 (SLC16A1) and MCT4 (SLC16A3) in the handling of lactate in cancer cells. Interestingly, even though it is easy to explain Warburg hypothesis by assuming all cancer cells behave as if they are in a hypoxic environment, it has become clear that cancer cells in solid tumors are heterogeneous, some clearly in hypoxic mode, but others in normoxic mode with ability for oxidative metabolism. This highlights the differential roles of MCT1 versus MCT4 in the handling of lactate. MCT4 seems to play a major role in the release of lactate from hypoxic cancer cells whereas MCT1 facilitates the uptake of lactate into oxidative cancer cells, thus providing a metabolic link and crosstalk between cancer cells in different microenvironment. This crosstalk might also extend to cancer-associated stromal cells.

Citrate is at the intersection of multiple biochemical pathways. It is a carbon source for endogenous synthesis of fatty acids and cholesterol, both occurring in the cytoplasm, and also a substrate for the Kreb’s cycle to generate ATP within the mitochondria. The article by Haferkamp et al. highlights the function of two different transporters for citrate, NaCT/SLC13A5 and pmCiC (an isoform of SLC25A1), both functioning in the plasma membrane, and their relevance to cancer. NaCT/SLC13A5 mediates the uptake of citrate from extracellular milieu into cancer cells (Jaramillo-Martinez et al., 2021) whereas pmCiC secretes citrate into the extracellular milieu from cancer-associated stromal cells (Drexler et al., 2021) but changes to a citrate importer in many cancer cell types (Mycielska et al., 2018), again underlining the metabolic crosstalk between different types of cells in solid tumors. Coleman and Parlo focus on cholesterol as an important metabolite for cancer-cell proliferation and describe the metabolic pathways for its endogenous synthesis using citrate as the carbon source. They also highlight the synthesis of citrate from glutamine via the cancer cell-specific pathway known as reductive carboxylation within mitochondria in which the reaction catalyzed by isocitrate dehydrogenase (isocitrate → α-ketoglutarate) is facilitated in the reverse direction in cancer cells by IDH2, an isoform of the enzyme that uses NADP^+^/NADPH instead of NAD^+^/NADH as the coenzyme.

Since cancer cells generate lactic acid and CO_2_ (i.e., H_2_CO_3_) in large quantities in metabolism, the cells have to find ways to eliminate the acid load and maintain intracellular pH. This is the topic of the review by Venkateswaran and Dedhar, primarily focusing on the CAIX isoform of carbonic anhydrase, a membrane-bound protein with its catalytic site located externally. This enzyme catalyzes the following reaction: CO_2_ + H_2_O → H_2_CO_3_ → H^+^ + HCO_3_^−^. The Na^+^/bicarbonate transporter NBCn1 (SLC4A7) then transports HCO_3_^−^ into cells, leaving H^+^ outside. CAIX and NBCn1 form a complex to carry out these functions efficiently.

We have seen in recent years an explosion of structural studies on membrane transport proteins aided by x-ray crystallography and cryogenic-electron microscopy. Many of these transporters are closely related to cancer-cell metabolism. Examples include SLC7A5, SLC1A5, SLC6A14, and SLC7A11. The article by Carusela and Rubi provides insights into the computational modeling of the structures of membrane transporters belonging to the ABC (ATP-Binding Cassette) family and the SLC (SoLute Carrier) family. With the ABC transporters, the authors highlight the structural changes that follow in connection with ATP hydrolysis. With SLC transporters, the focus is on the structural changes that follow the binding of substrates to the substrate-binding site.

It is well recognized that we need less-invasive biomarkers to diagnose different cancers and to monitor therapeutic efficacy, but success in this area still remains elusive. Two articles in this Special Issue (Du et al.; Ge et al.) use metabolomic profiles in serum as biomarkers for papillary thyroid cancer and intravenous leiomyomatosis, which pinpoint altered metabolism of aspartate and glutamate as well as the Kreb’s cycle and urea cycle in the former and the utility of hypoxanthine, acetylcarnitine, and glycerophosphocholine in the diagnosis of the latter.

### Synopsis

The articles assembled in the Special Issue collectively provide valuable insight into the cancer cell-specific reprogramming of metabolic pathways and the mechanisms by which the cancer cells acquire key nutrients to support their energy needs and promote their growth, proliferation, invasion and migration. These aspects of cancer-cell biology are fundamental for cancer progression and metastasis, thus fulfilling the primary goal of the Special Issue.
